# Giving voice to employees in low-skilled jobs works: Effect and process evaluation of a participatory sustainable employability intervention

**DOI:** 10.3233/WOR-230507

**Published:** 2024-12-16

**Authors:** Emmelie Hazelzet, Hans Bosma, Angelique de Rijk, Inge Houkes

**Affiliations:** Department of Social Medicine, CAPHRI Care and Public Health Research Institute, Faculty of Health, Medicine and Life Sciences, Maastricht University, Maastricht, The Netherlands

**Keywords:** Employees in low-skilled jobs, evaluation, mixed-methods, job control, sustainable employability, intervention

## Abstract

**BACKGROUND:**

To improve the sustainable employability (SE) of employees in low-skilled jobs, there is an urgent need to implement more effective approaches for this group.

**OBJECTIVE:**

This evaluation study aimed to get insight into the effect and implementation process of an organisational intervention called ‘Healthy HR’ (HHR), which promoted the job control and SE of employees in low-skilled jobs in two Dutch organisations.

**METHODS:**

An effect evaluation with a pretest-posttest design and a mixed-methods process evaluation were conducted. Quantitative data were collected at baseline (*N* = 120) and at 12 months’ follow-up (*N* = 71). Paired *t*-tests and dose-response analyses were performed (*N* = 50). Mixed-methods process data were collected on the implementation process using questionnaires, individual interviews with employees and employer representatives (*N* = 26), focus groups (*N* = 4) and logbooks.

**RESULTS:**

A positive effect was found for job control at 12 months’ follow-up. An effect on the distal outcome SE was not significant. The dose-response analysis showed that a higher dose of HHR resulted in better job control. This positive effect was supported by the qualitative process analysis. HHR had a positive impact on the awareness level about health and healthy workplaces among all stakeholders.

**CONCLUSIONS:**

This study showed a promising participatory approach to improve job control for employees in low-skilled jobs by actively involving them in a genuine dialogue and giving them an active voice. Effects on SE might require a longer follow-up.

## 1 Introduction

For many employers, sustainable employability (SE) is a topical challenge [1, 2]. It is increasingly acknowledged that SE and related concepts are beneficial for both employees and employers [3]. SE can be defined in a variety of ways but employee health, productivity and a valuable work context throughout employees’ working lives are core components of most definitions [1, 4]. Addressing employees’ SE via the work setting can potentially contribute to reducing socio-economic health inequalities [5, 6]. Although numerous organisational interventions have been developed and showed modest effects on employees’ health [7–10], many organisations are still unsuccessful in promoting employees’ SE [2].

In practice, difficulties can be observed particularly with regard to employees in low-skilled jobs. They hardly participate in workplace health promotion interventions, most likely due to a mismatch between what they need, their demanding job tasks and the top-down measures proposed and implemented [11, 12]. Moreover, this group often experiences adverse working conditions [11, 13, 14], which negatively impact their health. A recent European survey showed that most high to extremely strained jobs can be found in sectors with many low-skilled jobs such as industry (29.3%), transport (41.8%), hospitality (32%) and construction (28.3%). The percentages of high strained jobs in sectors with many high skilled jobs such as financial services (15.3%) and public administration (24%) are lower. The more strained the jobs, the higher the share of workers with accumulated health problems (46%) and emotional and physical exhaustion (31%) [15]. It is therefore difficult to reduce health inequalities given those conditions [11, 16, 17].

More insight is needed into the extent to which organisational interventions aimed at promoting SE are effective for employees in low-skilled jobs, who often are an underrepresented group in the field of occupational health [18–20]. Low-skilled jobs are characterised by high job demands and low job resources [11]. In the current study we focus on employees with lower and middle levels of education performing routine production tasks that do not require high-level specialised skills. For instance, in the Netherlands although the overall educational level is increasing, the majority (59%) of the working population is still categorized as lower educated [21]. An important condition for a successful organisational intervention is a participatory approach [22], in which there is active involvement and an active voice from employees in the development of such interventions [20]. Previous studies have shown that this participatory approach was effective with regard to health-related outcomes [14, 23]. Moreover, Peters, Nielsen, et al. [24] showed that when employees participate in the development and implementation of the intervention, they experienced an increase in ownership. This might result in an improvement of their perception of control at work and of their health. Job control refers to an employee’s ability to influence his or her work environment and to participate in decision making on the job [25]. This is an important resource to improve the overall health of employees, particularly for employees in low-skilled jobs who generally work in contexts of low control of their work [11, 25, 26].

To improve employees’ job control and eventually SE, a dialogue-based organisational intervention, ‘Healthy Human Resources’ (HHR) was developed, together with employees and employer representatives of five Dutch organisations, and researchers [27]. HHR implies a constant dialogue between employer and employees. Employees are stimulated to actively participate to develop and implement their own tailored solutions and are given an active voice in the process. We expect that this will lead to a higher perceived job control, which will eventually contribute to higher SE. The assumption is that active involvement and true dialogue will be activated when employers and employees systematically follow the stepwise approach in HHR [27].

We started to implement HHR in the five organisations involved in the development process, but found that none of them implemented it fully. A qualitative study revealed a laborious implementation process, barriers rooted in steep hierarchies, and a manifest lack of decision authority on the part of the middle-managers[28]. Based on these results, we slightly adapted the implementation process of HHR (e.g. more external consultation from researchers and fully dedicated project leaders) and proceeded with the implementation of HHR on a smaller scale.

**Fig. 1:**
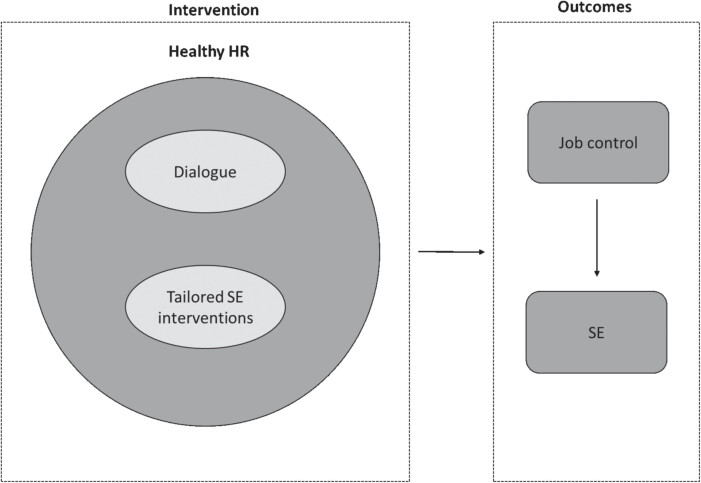
Conceptual model HHR (23).

The current study aimed to evaluate the effectiveness and implementation process of HHR on the job control and SE of employees in low-skilled jobs in two Dutch organisations. We formulated the following hypotheses for the effect evaluation: 1) the use of HHR will increase job control *(hypothesis 1a)* and eventually the SE of employees in low-skilled jobs (*hypothesis 1b*); and 2) a higher dose of HHR leads to greater job control (*hypothesis 2a*) and eventually a better SE among employees (*hypothesis 2b*) compared to a lower dose of HHR. The conceptual model of HHR is depicted in Fig. 1. The aim of the process evaluation was to get insight into the implementation process of HHR and support the understanding and interpretation of the effectiveness of HHR on job control and SE.

## 2 Methods and materials

**Fig. 2:**
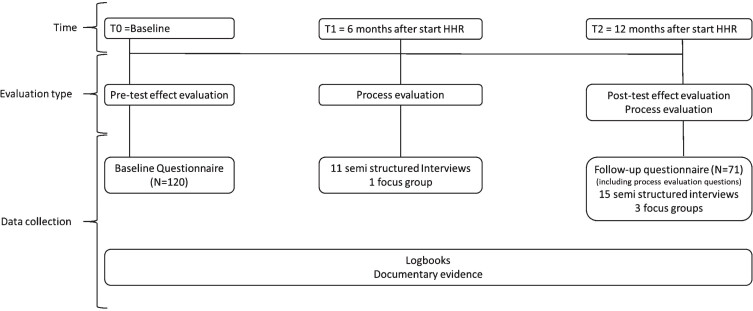
Overview evaluation moments and data collection methods of HHR.

The methods and data collection procedures have been explained in a protocol study [29] and a validation study of the adapted Maastricht Instrument for Sustainable Employability (MAISE-Easy) for measuring the outcome SE [30]. The MAISE-Easy appears to be a reliable and valid measurement instrument for measuring aspects of SE in employees who work in low-skilled jobs. Figure 2 presents an overview of the evaluation moments and data collection methods. This evaluative study was part of a larger research project including multiple studies [31]. For the development of MAISE-Easy, experts were consulted. In the development process of Healthy HR, employees and employer representatives were considered as primary experts from the practical field. This development process was supported by researchers from different disciplines (sociology; organizational psychology and occupational health). Data for the current study were collected through MAISE-Easy, interviews with employees and employers, and logbooks (also see Sections 2.3 and 2.4). This study was approved by the Medical Ethics Committee of the Academic Hospital in Maastricht, The Netherlands (METC 2017-0311), and all participants signed a written informed consent form before participating.

### 2.1 Study population & setting

The two Dutch organisations, including their employees, were recruited via the researchers’ personal network at the end of 2020. In the first one *(organisation A)*, a large technical service company (total size: 5500 employees), two regional departments were selected: A1 consisted of employees in technical modifications and assembly (*N* = 38), and A2 consisted of employees working in technical services and maintenance (*N* = 73). The second one *(organisation B)*, a production company, consisted of employees (*N* = 43) spread over three departments: B1) office, B2) production site and B3) assembly at the customer site. Employees were included when they performed low-skilled job, mostly with a lower level of education and spoke the Dutch language.

### 2.2 Intervention Healthy HR

HHR is an online toolkit that supports middle-managers with developing and implementing SE interventions together with their employees through a dialogue-based approach. They follow seven steps with these titles: (1) ‘Prepare together’; (2) ‘Measuring is knowing’; (3) ‘Our problems’; (4) ‘Our solutions’; (5) ‘Action plan’; (6) ‘Let’s start’; and (7) ‘Evaluation & embedding’. Each step consists of one or more instructions for tasks. Dialogue-based tools, such as working formats, checklists and fill-in templates, support the performance of each task. The tools facilitate active involvement and dialogue between employees and employer.

During the first step a project group is set up, consisting of a project leader, supervisor(s) and 3–4 employees’ representatives. The project leader facilitates HHR and chooses which tools fit best to their employees and organisational context. More details about the content and development of HHR have been reported elsewhere [27].

### 2.3 Effect evaluation

The effectiveness of the intervention was evaluated with a pretest-posttest design with a one-year follow-up. Data were obtained from employee questionnaires at T0 (baseline) and at T2 (12 months). The baseline questionnaire was distributed between February 2021 and May 2021.

#### 2.3.1 Outcome measures

*Job control *was measured by means of a self-developed scale consisting of five items (Cronbach’s alpha: 0.81). The items were inspired by existing scales, such as the Dutch Questionnaire on the Experience and Evaluation of Work and the Maastricht Autonomy Questionnaire [32, 33]. The response scale ranged from 1 (never) to 5 (always). Job control was used in two ways: 1) as a separate primary outcome measure in line with the conceptual model and 2) as a subscale of valuable work, one of the core components of SE (see below).

The measurement of *sustainable employability (*SE) was based on the defined SE core components (health, productivity and valuable work) found in an earlier study [4]. SE was measured by the health, productivity and valuable work scales, following the earlier protocol [29] and the validation study of the questionnaire used, the adapted Maastricht Instrument of Sustainable Employability (MAISE-Easy) [30]. The overall construct validity, reliability and criterion validity were adequate to good. *Health* was measured by the four items of the health scale from MAISE-Easy (Cronbach’s alpha: 0.62). The response scale ranged from 1 (never) to 5 (always). *Productivity* was measured by the five items from MAISE-Easy (Cronbach’s alpha: 0.74). The response scale ranged from 1 (never) to 5 (always). *Valuable work* consisted of the subscales *social work climate* (four items; Cronbach’s alpha: 0.79), *self-efficacy* (five items; Cronbach’s alpha: 0.77) and *job control* (as described above) from MAISE-Easy. Job control was analysed as a separate outcome (in accordance with the hypotheses), but also incorporated in the *valuable work* scale. The response scale ranged from 1 (never) to 5 (always) [30]. An average score was calculated for the health, productivity and valuable work scales to construct SE.

#### 2.3.2 Dose received

In the follow-up questionnaire, the dose received by employees was measured by means of 18 self-developed items representing the intervention tasks within the seven HHR steps (Table 5). Employees indicated whether they had become familiar with each intervention task. An example item for a task in HHR step 1 was: “I know who is in the working group of the Healthy HR”. A 3-point response scale was used (Yes, No, I don’t know). Percentages of ‘yes’ per item, the sum score of the items answered with ‘yes’, and the average sum score were calculated to indicate the dose received. Theoretically, the sum score ranges from no dose (=0) to full dose (=18).

#### 2.3.3 Data analysis

Data were analysed using IBM SPSS Statistics version 27. Paired *t*-tests were performed to assess changes in the outcome variables between T0 and T2 *(hypotheses 1a and 1b)* (*p*-value < 0.05 and <0.10). Subsequently, change scores were computed between T0 and T2 for the dose received-response relationship. Data were normally distributed. Pearson correlation coefficients were computed per organisation and separately per department to examine whether a higher dose of HHR received by employees was related to improved job control and SE (*hypotheses 2a and 2b*).

### 2.4 Process evaluation

A mixed-methods design was used for the process evaluation. We measured the following Linnan and Steckler’s process indicators [34]: recruitment, reach, dose delivered (completeness), dose received, fidelity (quality), satisfaction and context. Additionally, proximate outcomes (immediately arising results) or the so-called perceived changes in this study were assessed [35]. Figure 2 shows the data collection process. Both quantitative (i.e. follow-up questionnaire (T2)) and qualitative process data (i.e. semi-structured interviews, focus groups, logbooks) were collected from employees and employers. In total, at six months (T1), 11 individual interviews and one focus group (*N* = 5) were performed and, at T2, 15 individual interviews and three focus groups (*N* = 5–7) (for more details, see S1 Appendix). The individual interviews were held face-to-face, online or by telephone, depending on practical reasons. Different stakeholders at all levels were selected to maximise the variety of perspectives on the intervention, for instance employees within and outside the project group. The focus groups were equivalent to the composed project groups. The general topic lists for T1 and T2 for the specific stakeholders can be found in S2 Appendix. Topic lists were tailored to the organisations, for instance, taking progress changes into account. Throughout the entire intervention period, researchers kept a logbook about events, progress, changes in the specific organisational context, telephone calls, and observations for each organisation. We also received the logbook of the organisations when available (only organisation B kept a logbook).

#### 2.4.1 Measurement – process indicators

*Recruitment *was assessed by the description of different approaches to recruit employees in the logbook and interview data from employees and project leaders at T1. *Reach* was assessed by the percentages of the employees who filled in the questionnaire at baseline indicated at T2. During the follow-up questionnaire at T2, employees were asked whether they were familiar with HHR. *Dose delivered* (completeness) referred to the extent to which HHR was actually delivered by the project leaders according to the intervention plan (at least 14 project group meetings). During the interviews at T2, the project leaders (the end user of HHR) were asked about which steps of the HHR toolkit had been delivered and which materials had been used. The logbook data of the researchers and the project leader (if available) were consulted. *Dose received* by the employees was assessed by the quantitative dose received measure from the effect evaluation at T2. During the interviews with employees at T1 and T2, more insights were gained about the dose of HHR received. *Fidelity* (quality) represents the quality of the implementation of HHR. During the interviews at T1 and T2, project leaders were asked to describe how they followed the intervention plan and whether they adapted aspects of the intervention. *Satisfaction* was measured by an overall satisfaction score of employees on a 10-point scale (1 = very dissatisfied; 10 = very satisfied) in the follow-up questionnaire at T2. The reasons for the satisfaction score were revealed in the interviews and focus groups of different stakeholders (employees, supervisor, project leader and higher management) at T2. The *contextual factors* (omnibus, discrete) and company history (i.e. barriers, facilitators) that affected HHR implementation or outcomes were assessed by using logbook, interview and focus group data at T1 and T2. *Perceived changes* were measured by means of a question in the follow-up questionnaire at T2: “What benefit did HHR bring you?” A predefined list of potential perceived changes was provided with a 5-point response scale (1 = totally disagree; 5 = totally agree) and dichotomised to agree or disagree (%). During the interviews at T2, all stakeholders were asked about perceived changes at the individual and organisational levels.

#### 2.4.2 Data analysis

Methodological triangulation was the basis for the data analysis because three different methods were used (questionnaire, interviews/focus groups and logbook). Descriptive statistics (i.e. percentages, means and standard deviations) were done, using SPSS version 27 for the process indicators: reach, dose received, satisfaction and perceived changes. The open-ended questions were coded manually. All interviews were audio-recorded and transcribed verbatim. The first author (EH) analysed the transcripts and logbook data from the general viewpoint of the practical steps from the Qualitative Analysis Guide of Leuven (QUAGOL) [36]. The data were read, and relevant process indicators were identified. Subsequently, the data were coded using qualitative data software, Nvivo program version 12. During several meetings with the other authors, the qualitative analyses were discussed, and common themes related to the process indicators, the implementation process and perceived changes were interpreted in line with QUAGOL [36].

## 3 Results

In total, 120 (78%) employees completed the baseline questionnaire, and 71 employees completed the follow-up questionnaire (46%). A total of 48 employees completed both (31%). Table 1 shows the overall response rate and the response rates per organisation. The study population consisted mainly of men with a fulltime permanent contract holding a secondary vocational educational level. The mean age of the employees was 48.8 years (Table 2).

**Table 1:** Response rate questionnaire

Organisation	Number of employees	Number of respondents at baseline (T0) (*n*, %)^#^	Numbers of respondents at follow-up (T2) (*n*, %) – effect evaluation section	Numbers of respondents at follow-up (T2) (%) process evaluation section	Number of respondents overall (filled out both questionnaires)^#^
Organisation A1	38	38 (100)	29 (76)	28 (74)	19 (50)
Organisation A2	73	48 (66)	16 (22)	16 (22)	10 (14)
Organisation B	43	34 (79)	26 (60)	27 (63)	19 (44)
Total	154	120 (78)	71 (46)	71 (46)	48 (31)

^#^filled out the whole questionnaire.

**Table 2:** Baseline characteristics respondents

	Total sample	Organisation A1	Organisation A2	Organisation B
**Gender (*n*, % **)
Men	126 (96.9)	38 (100)	51 (94.4)	37 (97.4)
Women	4 (3.1)	–	3 (5.6)	1 (2.6)
Age (M)	48.8	47.9	49.2	49.3
**Educational level^**#**^ (*n*, % **)
- PS/Did not finish school	5 (3.9)	1 (2.7)	–	4 (10.5)
- LSE, SSE, SVE 1, SVE 2	57 (44.5)	16 (43.2)	15 (28.3)	26 (68.4)
- SVE 3-4	47 (36.7)	13 (35.1)	29 (54.7)	5 (13.2)
- HPE, University	19 (14.8)	7 (18.9)	9 (17.0)	3 (7.9)
**Type of contract (*n*, %)**
Fulltime-permanent	112 (86.2)	32 (84.2)	48 (88.9)	32 (84.2)
Fulltime-temporary	6 (4.6)	2 (5.3)		4 (10.5)
Parttime-permanent	8 (6.2)	1 (2.6)	6 (11.1)	1 (2.6)
Others^##^	3 (2.4)	2 (5.2)		1 (2.6)

^#^PS=Primary School, LSE = Lower Secondary Education, SSE = Senior Secondary Education, SVE = Secondary Vocational Education, HPE = Higher Professional Education. ^##^Fulltime-contract via employment agency; zero hours contract; secondment.

### 3.1 Effect evaluation

Table 3 presents the means and standard deviations of the baseline and follow-up measurements and results of the paired *t*-tests. With respect to the total sample, job control increased, while no significant change was found for SE. Productivity, one of the SE components, decreased. For both organisations separately, similar results were found, with a positive effect on job control observed in organisation B. Therefore, hypothesis 1a was confirmed, while hypothesis 1b could not be confirmed.

**Table 3:** Mean differences before-after and paired t-tests

	Organisation A1–A2 (*N* = 29)			Organisation B (*N* = 21)			Total sample (*N* = 50)		
	Before	After		Before	After		Before	After
	M (SD)	M (SD)	*p*	M (SD)	M (SD)	*p*	M (SD)	M (SD)	*p*
**Job control (1–5)**	3.33 (0.7)	3.41 (0.7)	0.512	3.19 (0.9)	3.41 (0.8)	0.028^*^	3.27 (0.8)	3.41 (0.7)	0.094^†^
**Sustainable employability (1–5)^**#**^**	3.84 (0.3)	3.73 (0.4)	0.167	3.74 (0.4)	3.77 (0.4)	0.669	3.80 (0.4)	3.75 (0.4)	0.363
Health (1–5)	3.92 (0.4)	3.79 (0.4)	0.138	3.62 (0.5)	3.70 (0.4)	0.477	3.80 (0.5)	3.76 (0.4)	0.569
Productivity (1–5)	3.94 (0.5)	3.70 (0.6)	0.044^*^	4.05 (0.6)	4.00 (0.6)	0.694	3.99 (0.5)	3.83 (0.6)	0.061^†^
Valuable work^##^	3.65 (0.5)	3.68 (0.5)	0.708	3.56 (0.5)	3.62 (0.5)	0.344	3.61 (0.5)	3.65 (0.5)	0.435
*Social work climate (1*–*5)*	3.77 (0.7)	3.66 (0.7)	0.398	3.60 (0.7)	3.48 (0.7)	0.366	3.70 (0.7)	3.58 (0.7)	0.218
*Self-efficacy (1*–*5)*	3.84 (0.5)	3.97 (0.7)	0.229	3.89 (0.5)	3.97 (0.6)	0.539	3.86 (0.5)	3.97 (0.6)	0.187

^*^p < 0.05; ^†^*p* < 0.10; Bold = variables for hypotheses; ^#^SE consists of health; productivity and valuable work; ^##^Valuable work consists of social work climate; self-efficacy and job control (depicted as a separate scale at the top of the table in accordance with the hypotheses, but also incorporated in the valuable work scale).

#### 3.1.1 Dose received – response relationships (H2a and H2b)

Table 4 presents the average dose received by employees and the dose received-response relationship. Overall, employees in organisation B reported the highest dose received. We found a significant positive association between dose received and job control in that organisation (0.43; *p* = 0.05). For B2 and B3 (the departments with the highest dose received), the correlation between dose received and job control was the strongest (0.68; *p* < 0.01). Regarding SE in these departments, the correlation with dose received was 0.29; this was, however, not statistically significant (*p* = 0.30). For organisation A, employees reported a lower dose received compared to organisation B, and no associations were found of dose received with either job control or SE. Hence, hypothesis 2a was confirmed, while hypothesis 2b could not be confirmed.

**Table 4:** Dose received and dose received-response relationship

	Dose-received employees (range: 0–18)^a^	Dose-received -response			
		Job control		SE
	M	*r*	*p*	*r*	*p*
Organisation A	6.3	–.07	0.725	–.09	0.638
A1 (*N* = 19)	7.8	–.16	0.496	–.30	0.221
A2 (*N* = 10)	3.8	.00	1.000	.08	0.825
Organisation B (*N* = 21)	9.3	.43	0.050^*^	.08	0.732
B1 (*N* = 6)	6.0	.48	0.340	–.17	0.749
B2–B3 (*N* = 15)	11.5	.68	0.006^**^	.29	0.300
Total sample (*N* = 50)	7.4	–.03	0.828	–.13	0.386

^*^p < 0.05; ^**^p < 0.01. ^a^see Table 5 for interpretation of the mean of dose-received.

**Table 5:** Overview process indicators per organisation

Process indicators	Organisation A1	Organisation A2	Organisation B
**Recruitment (N)**	+	+	+
External meeting (with researchers)	2	1	2
Internal kick-off meeting (without reseachers)
Higher management	1	1	1
Direct supervisors	1	1	1
Project group	1	1	1
**Reach (% yes)**	+	+/–	+
Familiar with HHR	86.2	74.1	100
Filled in baseline questionnaire	86.2	55	92.6
**Dose delivered (N)**	+	+	+
Number of HHR steps delivered by project leader	7	7	7
Project group meetings (min. 14)	7	7	14
Meetings all employees	7	7	1
**Dose received**	+/–	–	+/–
Average HHR steps received by employees	7.8	3.8	9.3
**Dose received per step (% yes)**
*HHR step 1* – *Prepare together (3 items)*
1. Information was given about what the project entailed by my supervisor/project manager	79.3	40.9	71.4
2. The purpose of the project was clear to me	82.8	40.9	71.4
3. I know who is in the project group of HHR	89.7	54.5	89.3
*HHR step 2* – *Measuring is knowing (3 items)*
4. I have completed the baseline questionnaire	86.2	55	92.6
5. The results of the questionnaire have been shared with us	58.6	40	48.1
6. The results of the questionnaire have been discussed	48.3	10	51.9
*HHR step 3* – *Problems (3 items)*
7. Information was given on the problems	48.3	15	59.3
8. I was able to brainstorm about ideas for the main problems	48.3	20	55.6
9. I could participate in decisions about the main problems	24.1	10	37
*HHR step 4* – *Solutions (4 items)*
10. Information was given on the solutions	31	11.8	55.6
11. I was able to brainstorm about ideas for the main solutions	31	17.6	63
12. I could participate in decisions about the main solutions	13.8	5.9	33.3
13. It was clear to me what solutions were chosen	20.7	0	40.7
*HHR step 5* – *Action plan (1 item)*
14. The plan was clear how and when the solutions were distributed on the work floor	17.2	0	37
*HHR step 6 and 7* – *implementation, evaluation* & *embedding (4 items)*
15. I see the chosen solutions reflected in the workplace	10.3	0	25.9
16. Information about the continuation of HHR was given	48.3	5.9	29.6
17. I was always informed about the steps and actions taken within HHR by my colleague/project leader/manager	24.1	17.6	44.4
18. I was always invited to express my opinion	20.7	17.6	25.9
**Fidelity**	+/–	+/–	+/–
Adaptations made	Yes	Yes	Yes
**Overall satisfaction^**# ;**^ M(SD)**	6.2 (1.3)	5.2(2.1)	5.6 (1.6)
Per department
Office			4.1 (1.6)
Production			5.9 (1.1)
Assembly			7.0 (1.3)
**Context^**##**^**
Organisational culture	B	B	B/F
Psychological safe environment	F	F	F
Communication	F	B	B
Pandemic	–	B	B
Practical resources (e.g. time, support)	B	B	F
National construction holiday period	–	–	B
**Perceived changes (% agree)**
I am more actively involved	20.7	11.8	26.9
I am having more conversations about a healthy workplace	27.6	17.6	26.9
My wishes are listened to more seriously	20.7	11.8	22.2
I can express my views on healthy work more often	48.3	23.5	38.5
I have better communication with supervisor	27.6	11.8	36
I have better communication with colleagues	37.9	17.6	52
My work environment has improved	3.4	5.9	20
I am more aware of my health	48.3	29.4	76.9
I feel more responsible for a healthy workplace	58.6	17.6	68

Note. + = good; +/– = moderate; – = low. ^#^Satisfaction was rated on a 1–10 scale (1 = very unsatisfied; 10 = very satisfied), only respondents included who are familiar with HHR. ^##^B = barrier; *F* = facilitator.

### 3.2 Process evaluation

Table 5 provides an overview of the findings with regard to all process indicators. Generally, HHR was experienced as more effective and better implemented in organisation B compared to organisation A. The seven different process indicators are addressed below, plus the perceived changes. Subsequently, we aimed to interpret the effect evaluation better using the insights from the process evaluation.

1. ***Recruitment***
  In organisation A, two project leaders were selected to facilitate HHR for organisation A1 and A2, respectively. Internal kick-off meetings were organised to inform participants about HHR and build support among higher management, direct supervisors and the already arranged project groups. The employees within the project groups were mainly recruited informally and invited by their direct supervisor to participate and represent their colleagues.
  In organisation B, one project leader was selected to facilitate HHR and organise internal kick-off meetings. The recruitment strategy was mainly informal, and employees were invited by their supervisor or the project leader. During the recruitment period, several internal meetings were organised to inform the higher management. The project leader was often on site and available to answer questions and to inform employees in more detail.
2. ***Reach***
  In organisation A, a majority of the employees was familiar with HHR and indicated that they filled in the baseline questionnaire. In organisation B, all employees (100%) were familiar with HHR, and almost all of them indicated that they filled in the baseline questionnaire.
3. ***Dose delivered (completeness)***
  In organisation A, all steps of HHR were delivered according to both project leaders, but the extent to which the steps, tasks and tools were followed varied. The tools for brainstorming, prioritising and voting were used, but rather as a means to an end: “*It’s about getting the information out there, and if it’s just not through that precise format, fine.*” *(Project leader, A2).* The communication tools were used, such as posters. In total, seven project group meetings were organised, which is fewer than indicated in the intervention planning.
  In organisation B, the first four steps of HHR were strictly followed, while the remaining steps were loosely followed, as explained by the project leader: “*We deviated from the toolkit, but a plan of action was made. We also did regular mid-term evaluations [...] step seven, we performed the final evaluation and we’re also working on embedding. We are doing it, only slightly differently than you had indicated.*” *(Project leader, B).* This also applies to the tools. At the start the communication tools were mainly used, while during the process they lost themselves in the action plan and forgot about the other tools in the HHR toolkit. In total, 14 project group meetings were organised, in conformance with the intervention planning.
4. ***Dose received***
  For organisation A, the dose received by employees decreased as the process progressed (Table 5). A lack of visibility of HHR played an important role. “*The disadvantage for the men is that they don’t see immediate results. They are, of course, people who are involved in the daily performance.*” *(Supervisor, A1).* The project group focused primarily on the long-term ‘behind the scene’ solutions, which were less tangible and not perceived by the other employees on the workfloor. Consequently, solutions were not directly associated with HHR: “*We achieved quite a good result with the solutions, only that the connection with HHR is difficult to make. So we should have been more visible.*” *(Project leader, A2).* This could be a reason for the gradually lower dose of HHR among employees throughout the process. The “healthy” aspect in the name HHR was perceived as misleading for higher management and project leaders, as they did not regard improving work conditions to be related to health. Employees acknowledged this relationship, however. One employee explained, “*Healthy HR, I actually see it this way, I think it’s broader than health. There’s also a piece of business administration in here alongside Healthy HR, so healthy is linked to progress and the way things are done at work.*” *(Assembly employee, A1).*
  For organisation B, a gap existed between employees who barely noticed the existence of HHR and employees who received the process of HHR: “*I didn’t notice or see anything of the whole process here in the office.” (Office employee, B)*. Miscommunication from the project group members to the rest of the employees (B1) in terms of framing the wrong message about the goal of HHR – already at the start – played an important role. Other employees received the process of HHR but experienced ‘little action’: “*More attention is being paid to it, but at this point I see little benefit.*” *(Assembly employee, B).* This illustrates a possible reason why the dose lessened from the end of HHR step 4. The visibility aspect was also observed in organisation B, who focused more on the ‘quick wins’ and ‘visible’ solutions: “*when they saw that a fair amount was happening, they began to write down more.*” *(Project group member production, B).* Hence, employees experienced that their voice was heard because of a higher visibility of their ideas and a higher dose of HHR, at least in B2 and B3.
5. ***Fidelity (quality)***
  In organisation A, no major deviations from the intervention plan were noted, but the way the project leaders followed and adapted HHR affected the quality of implementation in a later phase. HHR was used as a leading principle. They felt prepared to facilitate the project group meetings and valued the HHR toolkit: “*Saves some time thinking about how you’re going to shape those sessions, or the step of how you’re going to structure that.*” *(Project leader, A2).* Several adaptations were made, such as a PowerPoint presentation for each project group meeting to give more guidance, tailoring of the communication tools and a tool to increase engagement.
  In organisation B, the project leader used HHR as a leading principle, and no major deviations were shown from the intervention plan until step 4. The project leader followed HHR much less towards the end of the implementation of the chosen solutions. Some adaptations were made which affected the quality of implementation positively. For instance, the project group representative of B2 placed a notebook in the production hall to collect ideas from colleagues. In B3, a WhatsApp group was introduced to transfer information about HHR among colleagues, because of their low presence in the main building due to their type of job.
6. ***Satisfaction***
  In organisation A, relatively low satisfaction among the rest of the employees was related to a low dose of HHR received on the workfloor. Project group members described a sceptical (“nothing will change anyways”) and disinterested attitude among a group of colleagues. One described a colleague’s reaction: “*I get my money every month and let me do my thing, don’t bother me.*” *(Employee project group, A1).* As the process continued, this negative attitude disappeared to some extent, and a more interested attitude emerged. The ‘positive vibe’, high engagement and responsibility of all project group members contributed to greater satisfaction. Although the employees in the project group were positive, they experienced difficulties in taking an active role in this participatory process. Project leaders were generally satisfied and experienced their facilitating role as both positive, challenging and time-consuming. The human resource director also appreciated HHR: “*The brainpower of the employees was actually used.*” *(HR director, A).*
  In organisation B, the feeling of accountability and being heard by their employer contributed to greater satisfaction, particularly among employees in B2 and B3. Low satisfaction among employees from B1 was due to less involvement and wrong communication at the start of HHR: “*Project would be relevant to mechanics and factory, not office.*” *(Office employee, B*). During the process, the feeling of responsibility declined among project group members, possible affecting the satisfaction of the rest of the employees. The project leader was generally satisfied but experienced her role as intensive and time-consuming. Her positive and pragmatic attitude contributed to a safe and open climate in which employees could share their ideas. The higher management also appreciated the process and recognised lower engagement from B1: “*Still alive, but not yet matured.*” *(Local boss, B).*
7. ***Context***
  In organisation A, a performance-based culture existed in terms of profit. Project leaders were challenged to demonstrate the value of HHR to higher management. Accordingly, higher management was more open to HHR. For organisation A2, only “narrowcasting” (tv-screen communication) was possible and hindered the communication transfer of HHR to employees. Combined with the COVID-19 pandemic, digital communication was required, which was not beneficial for this group of employees. Organisation A1 primarily used the physical monthly meetings to inform all employees about HHR, which was positive to keep HHR ‘alive’ and visible. Employees in the project group were challenged to inform their colleagues. This depended on the project-based nature of their work at customer locations and how many colleagues they could speak to. The open and safe environment within the project groups acted as a facilitator, resulting in highly engaged project group members.
  In organisation B, the organisational culture was transforming from a traditional hierarchical culture (barrier) to a ‘learning organisation’ culture (facilitator), meaning that the employees were encouraged and stimulated to express their own ideas and build autonomy. The traditional culture was still evident among the older employees (>50 years and above) in terms of the feeling of broken promises and not being taken seriously by their old boss. Resistance to change and not participating in HHR were more common among them than among the younger employees. “*The previous boss did nothing about safety and health. Sometimes things were promised, and now the men have the feeling that something is being done. They still have to learn that a little bit. “(Production employee, B).* Ad hoc communication and a lack of it by several project group members to the rest of the employees and higher management negatively affected the process, causing stagnation. The positive communication skills of the project leader often counterbalanced this. The COVID-19 pandemic further negatively affected the implementation of HHR in terms of lower dose and engagement: “*COVID-19 could also have been a disruptive factor, people have not always been present at the times when something may have been shared.*” *(Office employee, B).* The psychological environment in terms of trust and openness within the project group was facilitating. Hierarchical power and an ‘us-versus-them’ relationships were not observed. Practical issues, such as a national construction holiday period and changes in project group members, delayed the implementation of HHR. However, enough time and support for the employees to participate in HHR acted as a facilitator.
8. ***Perceived changes***
  The majority of the employees in organisation A1 were more aware of their own health, and felt more responsible for a healthy workplace. In organisation A2, employees experienced significantly fewer perceived changes, because of the low dose of HHR received. The start of a change was commonly perceived by different stakeholders: “*This project sets tongues wagging, in a positive sense.*” *(Project group member employee assembly, A2).* Additionally, more awareness was experienced, but some employees agreed that changes were too early to notice because of the short duration of the project. The project leaders’ empathy skills improved because of HHR: “*There is now a real picture of what motivates these men, so to speak. And I think that’s actually the best achievement, because I think ultimately within the organisation, the thinking is very often done for people, without them (the men) really being heard and seen.” (Project leader, A1*). The project leader of organisation A2 confirmed her improved communication and support skills towards this specific group of employees. HHR requires these skills to understand ‘*the language that these men speak*’. Awareness about the dialogue and human aspect were perceived: “*It has resulted in us talking about it now, that at the end of the day we have to look to the human being even more [* ...  *] that we have included job satisfaction, collaboration, spirit as part of the annual plans.” (Supervisor, A1).* Furthermore, the higher management’s awareness improved because of HHR: “*By the fact that it has been talked about, that it has come to the attention of higher management, then you see that they are more open for listening.*” *(Supervisor, A1)*.

In organisation B, the majority of the employees experienced better communication with colleagues, were more aware of their own health, and felt more responsible for a healthy workplace. Employees of B2 and B3 experienced a “collective voice” and more awareness: “*I now have more of a feeling that you can achieve something because you and the group are strong, there is HHR in the company. So we are actually all consciously working on it a bit anyway.” (Project group member assembly, B)*. However, the employees of B1 perceived fewer changes because of the wrong communication about the expectations and hence the low dose received. The project leader experienced a personal learning curve in terms of improved communication and motivational skills towards employees. At the organisational level, she recognised the improved dialogue: “*I think for the company that they are now really getting that input from that employee, at least from assembly and from production. That they’ve really found a way that they can talk with people.” (Project leader, B).* Higher management also experienced this: “*You notice that people are quicker now to talk about something, and that wasn’t the case before.” (Boss, B).*

### 3.3 Interpretation of effectiveness in light of the process evaluation

The process evaluation showed that the positive effects of job control found in organisation B can be understood by better implementation of HHR. There were more project group meetings, a higher dose received by employees in specific departments, a higher visibility of HHR, and the organisational culture was more facilitating. The majority of the employees reported that they had been informed and had been able to brainstorm together about the problems and solutions. Participation in the actual decision-making was experienced less often. Overall, employees experienced a “collective voice”, more awareness and responsibility for their health and a healthy workplace. Focusing on quick-win solutions contributed to a higher visibility of HHR among employees. Additionally, by stimulating autonomy this contributed to a different mindset already being established within the organisation prior to the implementation of HHR.

For organisation A, no effect on job control was found, which can be explained by a lower implementation. There were fewer project group meetings, a lower dose received by employees, and lower visibility of HHR and engagement due to communication barriers. The implementation of HHR was mainly done and positively perceived by members of the project group, and difficulties were encountered in communicating effectively with the rest of the employees. In organisation A2, the received dose deteriorated after HHR step 2, while in organisation A1 the received dose of HHR became less after HHR step 3. As a result, the visibility and engagement among the rest of the employees faded away.

For both organisations, the process evaluation did not offer further insight into the negative effect on productivity and the lack of effect on SE.

## 4 Discussion

This study evaluated the effectiveness and the implementation process of an organisational intervention called ‘Healthy HR’ (HHR) to promote the job control and SE of employees in low-skilled jobs. An effect evaluation was conducted with a pretest-posttest design and a one-year follow-up among employees (*N* = 50) in two Dutch organisations. In parallel, a mixed-methods process evaluation was performed following the Linnan and Steckler’s process indicators. Additionally, the dose received-response relationship was tested.

Overall, effects were found on job control but not on SE. The implementation of HHR varied, as is often found. Previous scholars showed that it is almost impossible to act on unintended consequences due to the interrelated systems and to implement an intervention exactly as planned [37]. The variation in degree of implementation allowed us to test the dose received-response relationships, and we found a relation between a higher dose received and increased job control in organisation B. The process evaluation revealed how this effect came about, a higher dose received of HHR among employees, a facilitating organisational culture and the experience of a ‘collective voice’ among employees appeared to play a role.

The ambiguous effect of HHR on the distal outcome SE might be explained by the fact that changing this outcome may require much more time [18, 38]. Previous scholars also agreed that SE is a complex multi-dimensional concept involving many components [4, 39]. SE is the result of an employee-job environment interaction rather than just a personal characteristic. Even in the more matured and evaluated workplace health promotion interventions, the focus is still on individual characteristics such as lifestyle, health, and short-term behavioural effects at individual level [5, 40]. SE interventions ought however to address all core components of SE and also focus more on long-term effects [4]. Changes in these types of outcome variables are more difficult to achieve.

The results with regard to job control and the perceived changes are very promising and considered to be the first steps in a chain of events which will ultimately also change distal outcomes such as SE [18]. Based on the positive, albeit statistically not significant, correlation (*r* = 0.29) between the dose received and SE in organisation B2 and B3, we expect that the effect would have been even stronger after prolonged and more extensive implementation.

We found a decrease in productivity in one of the organisations that is difficult to interpret. The measure we used does not reflect productivity measured in terms of presenteeism and absenteeism [41], but a more global employee experience. While the quantitative analysis revealed a decline in productivity, this was not supported by the qualitative process analysis: none of the interviewees shared the impression of lowered productivity. Still, HHR might have cost time that could not be invested in the core work tasks. Alternatively, employees might have become more aware of and felt more responsible for productivity in relation to their work tasks due to extensive discussions on the topic in the project groups. Consequently, they might have been more critical about their own productivity at follow-up. A response shift might thus have occurred. Another explanation could be a ‘ceiling effect’ [42]. The baseline score of the total sample for ‘productivity’ was high (3.99 on a 5-point scale), and an already relatively ‘good’ score makes a significant increase less likely.

This study showed a promising participatory approach to improve the commonly low job control of a group [14, 16] that is underrepresented in occupational health research. Improving job control seems a good starting point, while achieving higher SE might require a longer follow-up. Job control as key – to actively involve employees in low-skilled jobs and give them an active voice via a continuous dialogue – may eventually improve their SE and might help to reduce health inequalities in the workplace [17].

### 4.1 Strengths, limitations and future research

The integration of the effect evaluation with the mixed-methods process evaluation is a strength of this study [43, 44]. The process evaluation with quantitative and qualitative data collection methods provided profound insights into the intervention process [45]. It supported performing a valid evaluation of the effectiveness and controlled for a type III error (i.e. concluding an intervention is ineffective when there is actually an implementation failure [43]). Data were collected at different levels to look at different stakeholder perspectives, including the higher management perspective [46]. Collecting qualitative process data throughout the process and after six months (T1) led to the creation of an improvement cycle by transferring the findings back to the organisations to support them in achieving the intended results. Measures for SE are scarce, particularly questionnaires that can be used among employees in lower-skilled jobs. In this study, we used the MAISE-Easy to measure SE; this is a new, self-developed questionnaire, adapted to employees in low-skilled jobs [30]. The SE measure consisted of three components (health, productivity and valuable work) with moderate to good internal consistency and validity [4, 30]. From a psychometric perspective, combining these three components into one uniform measure for SE appeared to be difficult. In the effect evaluation we decided to analyse the components (subscales) of SE separately as well. Further exploration and validation of the MAISE-Easy is needed to improve the measurement of SE in future research.

The limitations in this study were the small sample size, the low response rate at follow-up, the absence of the traditional control group and a short follow-up period. First, multiple questionnaires are a burden for most organisations, and particularly a lower dose of HHR is received among employees, this resulted in a lower response and a smaller sample size at follow-up. The reliance on people filling in the questionnaires should be taken into account [47]. Second, the use of a control group is often not feasible in complex organisations. The lack of the control group was counterbalanced by the dose received-response analyses and the mixed-methods process evaluation, which have also been used in other studies [38]. Third, a longer follow-up period is recommended to execute and imbed HHR because of the promising results of the proximate and intermediate outcomes. A short follow-up period makes full institutionalisation into existing organisational processes and individual behaviour almost impossible. In particular, the first four steps of HHR are relatively time-consuming to execute because of the high level of dialogue and need to obtain a complete picture.

### 4.2 Practical implications

HHR appears to be an effective approach for the improvement of job control of employees in low-skilled jobs. However, it can be disruptive and challenging for organisations as it requires another mind-set of all involved stakeholders [28]. Contextual and behavioural pre-conditions exist, such as a good fit with the organisational structure and culture, positive attitude and intrinsic motivation, good communication, patience from all parties and time to change. Implementation needs to be supported by external consultants, including monitoring and developing a tailored communication plan, even more intensely than what was offered during this study. HHR had initially been introduced as a self-led organisational intervention, but this study showed the positive effects of more stimulation and support.

With respect to persistence, a long-term process of implementation and embedding of HHR in organisational structures and processes is required to make it into a self-evident routine and to promote SE. The results showed that diverse project leader skills are needed or should be developed to effectively engage and guide employees in low-skilled jobs in this participatory process to create a safe and open environment, facilitating a health promoting culture [20, 48, 49]. Throughout the process, the visibility of HHR is extremely important for employees in low-skilled jobs to enable them to be engaged and willing to change. Showing the quick wins and the progress of HHR will help to keep employees on board.

## 5 Conclusion

The present study aimed to gain insight into the effect and implementation process of an organisational intervention called ‘Healthy HR’ (HHR) to promote job control and SE of employees in low-skilled jobs. HHR had a positive effect on job control, while SE was not improved during the 12 months’ follow-up. A higher dose received of HHR resulted in better job control. The process evaluation showed that lower implementation related to a lack of effect. By actively involving employees in low-skilled jobs in a continuous dialogue by giving them an active voice – thus through a participatory approach –, this study demonstrated how the job control of these employees can be improved successfully. Future evaluation research on SE should focus on a better implementation strategy and a longer follow-up.

## Ethical approval and informed consent

This study was approved by the Medical Ethics Committee of the Academic Hospital in Maastricht, The Netherlands (METC 2017-0311), and all participants signed a written informed consent form before participating.

## Conflict of interest

The authors declare that they have no conflict of interest.

## Supplementary Material

Supplementary File 1

Supplementary File 2

## Data Availability

The datasets generated and analysed during the current study are not publicly available due to the personal and sensitive information from the involved organisations and their participants (representatives of employers and employees). The data might be traced back to the organisations and individual respondents. The data is only available after contact with the corresponding author on reasonable request.

## Supporting information

S1 file. Overview number of interviews per organisation.

S2 file. Topiclists.
