# Supplementary material for: Giving voice to employees in low-skilled jobs works: Effect and process evaluation of a participatory sustainable employability intervention
Source: Work. 2024 Dec 16;79(4):1851–66. doi: 10.3233/WOR-230507 (PMC11664181; doi:10.3233/WOR-230507)
Supplement: Supplementary File 1 [file wor-79-wor230507-s001.docx]

S1 Appendix

Overview number of interviews per organisation

|  | **T1** | **T2** |
| --- | --- | --- |
|  | Number of interviews/focus groups | Number of interviews/focus groups |
| Organisation A1 | Individual interviews:  2 employees  1 project leader  1 supervisor | Individual interviews:  2 employees  1 project leader  1 supervisor |
|  |  | 1 Focus group:  3 employees  1 project leader  1 supervisor  2 team leaders |
| Organisation A2 | Individual interviews:  2 employees  1 project leader | 1 Individual interviews:  1 project leader |
|  |  | 1 Focus group:  3 employees  1 project leader  1 manager |
| Organisation B | Individual interviews  3 employees  1 project leader | Individual interviews:  6 employees  1 project leader  2 local bosses  1 CEO holding |
|  | 1 Focus group:  1 project leader  3 employees  1 manager | 1 Focus group:  4 employees  1 project leader |
| Total |  |  |
| Individual interviews | 11 | 15 |
| Focus groups | 1 | 3 |
