# Supplementary material for: Giving voice to employees in low-skilled jobs works: Effect and process evaluation of a participatory sustainable employability intervention
Source: Work. 2024 Dec 16;79(4):1851–66. doi: 10.3233/WOR-230507 (PMC11664181; doi:10.3233/WOR-230507)
Supplement: Supplementary File 2 [file wor-79-wor230507-s002.docx]

S2 Appendix

**Topic list 1 – individual interview of employees on the workfloor at T1**

**Introduction**

- Goal of interview: We are halfway through with Healthy HR (six months), we would like to see how the process is going and what your experiences are so far with Healthy HR.
- Practicalities: recording, informed consent form
- Introduction round

**Experiences with Healthy HR**

- Can you share what you think Healthy HR is about? What is the goal (fidelity)?
- What do you think of Healthy HR so far (satisfaction)?
- What was your reason for participating?

**Process**

- Can you talk about what has been done around Healthy HR so far? What have you noticed in terms of activities? (dose received)
- How were you informed/how did you get information about Healthy HR? In the beginning? (recruitment)

***Involvement and dialogue***

- How were you involved in the beginning? And now? (recruitment)
- Do you feel that you can think and decide about your job? (dose received) How do you feel about this?
- Do you feel that you can express your opinions and that they are listened to?

**Perceived changes**

- Are you already noticing a change in the workplace? Or within yourself? Could you describe this?

**Closing**

- Are there any other things you would like to share with us?

**Topic list 2 – focus group with project group at T1**

**Introduction**

- Goal of interview: We are halfway through with Healthy HR (6 months), we would like to see how the process is going and what your experiences are so far with Healthy HR.
- Practicalities: recording, informed consent form
- Introduction round

**Experiences with Healthy HR**

- What is your experience with Healthy HR so far (satisfaction)?
  - What are the strengths of Healthy HR?
  - What could be improved?

**Process**

- Can you tell us about the process so far? (dose delivered)
  - In the beginning?
  - What activities have been done?
  - What difficulties have been encountered?
- Can you talk about your role in Healthy HR? How did you approach it?
- What result have you achieved so far?

**Involvement and dialogue**

- How are employees informed/how do employees get information about Healthy HR? (recruitment)
- In what ways did you engage employees in the beginning? And now? To what extent did you experience difficulties?
- How did employees react to Healthy HR?
- To what extent does everyone participate and can everyone think and decide for themselves?

**Perceived changes**

- Are you already noticing a change in the workplace? Or within yourself? Could you describe this?

**Closing**

- Are there any other things you would like to share with us?

**Topic list 3 – individual interview with project leader at T1**

**Introduction**

- Goal of interview: We are halfway through with Healthy HR (six months), we would like to see how the process is going and what your experiences are so far with Healthy HR.
- Practicalities: recording, informed consent form
- Introduction round

**Experiences with Healthy HR and process**

- What is your experience with Healthy HR so far? (satisfaction)
- Can you describe the vision/purpose of Healthy HR?
- How do you think employees have responded to Healthy HR?
- How does the Healthy HR toolkit help to engage and implement the dialogue with employees? (fidelity)
- How did you make sure to engage employees?

**Healthy HR Toolkit**

- How does the Healthy HR toolkit help you?
- How do you use the toolkit? (fidelity)
- To what extent have the steps of the Healthy HR toolkit been implemented so far? (dose-delivered)
- Are there issues you have run into with the Healthy HR toolkit or the process? factors?

**Perceived changes**

- Are you already noticing a change in the workplace? Or within yourself? Could you describe this?

**Closing**

- Are there any other things you would like to share with us?

**Topic list 1 – individual interview of employees on the workfloor at T2**

**Introduction**

- Goal of interview: We are at the end of Healthy HR, we would like to discuss your experiences from the last six months.
- Practicalities: recording, informed consent form
- (If needed) introduction round

**Experiences with Healthy HR**

- What is your experience with Healthy HR? How was the process? Were you satisfied? Why were you satisfied/not satisfied?
- What was your reason/motivation for participating?

**Process**

- What have you noticed in terms of activities around Healthy HR? What components have you participated in? (dose received)
- How were you informed/how did you receive information about Healthy HR?
- How were you involved?
- Did you feel you were allowed to think and decide for yourself? (dose received) How do you feel about this?
  - If yes, what caused this? How did this happen?
  - Has it changed because of Healthy HR? Or was it always this way? Has management also changed because of this?
- Do you feel you can express your opinion openly and freely?

**Perceived changes**

- Has Healthy HR led to a change in the workplace/yourself?
- If Healthy HR continues, are there things you would do differently? Any tips?

**Closing**

- Are there any other things you would like to share with us?

**Topic list 2 – focus group with project group at T2**

**Introduction**

- Goal of interview: We are at the end of Healthy HR. We would like to discuss your experiences from the last six months.
- Practicalities: recording, informed consent form
- Introduction round

**Experiences with Healthy HR**

- In retrospect, what is your experience with Healthy HR (satisfaction)?
  - What are the strengths of Healthy HR?
  - What could be improved?

**Process**

- Can you share how the process went? (dose delivered)
  - Influencing factors? Did you feel there were any obstacles? (leadership?)
  - What went well/not well?
  - What activities were done? Which materials were used?
- How did you perceive your role in the working group? How did the collaboration go? Were you helpful to each other?
- What result did you achieve?

**Involvement and dialogue**

- How were employees informed/how did employees receive information about Healthy HR?
- How did you continuously engage employees? Interaction? What kind of materials were used? Fixed time in the week?
- To what extent did everyone participate and could everyone think? have a say? Did you feel there were barriers?

**Perceived changes**

- Has Healthy HR led to a change in the workplace/yourself?
- If Healthy HR continues, are there things you would do differently? Any tips?
  - What do you need?

**Closing**

- Are there any other things you would like to share with us?

**Topic list 3 – individual interview with project leader at T2**

**Introduction**

- Goal of interview: We are at the end of Healthy HR. We would like to discuss your experiences about the last six months, the process and the toolkit.
- Practicalities: recording, informed consent form
- Introduction round

**Experiences with Healthy HR**

- In retrospect, what was your experience with Healthy HR?
  - What did you find easy?
  - What did you find difficult?
- What factors influenced the implementation of Healthy HR? What facilitated it? Did you feel there were barriers?
- How satisfied are you with Healthy HR and how it went?
- Did Healthy HR help to establish a dialogue with employees and constantly engage them?

**Healthy HR Toolkit**

- Overall, what do you think of the toolkit?
- How did you use the toolkit?
- Did you implement Healthy HR as planned? If not, what have you done or how have you modified things differently (which ones and why)?
- What parts of the toolkit were delivered to employees?
- What tools did you use? Were they useful/not useful with the target group?
- Did the Healthy HR toolkit help you?

**Perceived changes**

- Did Healthy HR lead to a change in the workplace?
- Did Healthy HR deliver something for yourself/for the company?
- If Healthy HR continues, are there things you would do differently? Any tips?
  - What do you need?

**Closing**

- Are there any other things you would like to share with us?

**Topic list 4 – interviews with higher management at T2**

**Introduction**

- Goal of interview: We are at the end of Healthy HR. We would like to discuss your experiences about the overall process.
- Practicalities: recording, informed consent form
- Introduction round

**Experiences with Healthy HR**

- In retrospect, what was your experience with Healthy HR?
  - What are strengths of Healthy HR?
  - What could be improved?
- What factors influenced the implementation of Healthy HR? What was facilitating? Did you feel there were barriers?
- How satisfied are you with Healthy HR and how it went?

**Process**

- Does Healthy HR help with employee engagement?
- -What role did you play in Healthy HR? How did the collaboration go?
- Did Healthy HR make you look at employee health differently? And has that influenced your leadership style?

**Perceived changes**

- Has Healthy HR led to a change in the workplace?
- Has Healthy HR delivered anything for yourself/for the company?
- If Healthy HR continues, are there things you would do differently? Any tips?
- Any plans for the future/follow-up?

**Closing**

- Are there any other things you would like to share with us?
